# Reflectance-Based Vegetation Index Assessment of Four Plant Species Exposed to Lithium Chloride

**DOI:** 10.3390/s18092750

**Published:** 2018-08-21

**Authors:** Nicole E. Martinez, Julia L. Sharp, Thomas E. Johnson, Wendy W. Kuhne, Clay T. Stafford, Martine C. Duff

**Affiliations:** 1 Department of Environmental Engineering and Earth Sciences, Clemson University, Clemson, SC 29631, USA; 2 Department of Statistics, Colorado State University, Fort Collins, CO 80523, USA; Julia.Sharp@colostate.edu; 3 Department of Environmental and Radiological Health Sciences, Colorado State University, Fort Collins, CO 80523, USA; thomas.e.johnson@colostate.edu; 4 Savannah River National Laboratory, Aiken, SC 29808, USA; wendy.kuhne@srnl.doe.gov (W.W.K.); martine.duff@srnl.doe.gov (M.C.D.); 5 Department of Anesthesia & Perioperative Medicine, University of South Carolina Medical School, Columbia, SC 29808, USA; claytstafford@gmail.com

**Keywords:** reflectance spectroscopy, lithium, vegetation indices, *Arabidopsis thaliana*, *Zea mays*, *Brassica napus*, *Helianthus annuus*

## Abstract

This study considers whether a relationship exists between response to lithium (Li) exposure and select vegetation indices (VI) determined from reflectance spectra in each of four plant species: *Arabidopsis thaliana*, *Helianthus annuus* (sunflower), *Brassica napus* (rape), and *Zea mays* (corn). Reflectance spectra were collected every week for three weeks using an ASD FieldSpec Pro spectroradiometer with both a contact probe (CP) and a field of view probe (FOV) for plants treated twice weekly in a laboratory setting with 0 mM (control) or 15 mM of lithium chloride (LiCl) solution. Plants were harvested each week after spectra collection for determination of relevant physical endpoints such as relative water content and chlorophyll content. Mixed effects analyses were conducted on selected endpoints and vegetation indices (VI) to determine the significance of the effects of treatment level and length of treatment as well as to determine which VI would be appropriate predictors of treatment-dependent endpoints. Of the species considered, *A. thaliana* exhibited the most significant effects and corresponding shifts in reflectance spectra. Depending on the species and endpoint, the most relevant VIs in this study were NDVI, PSND, YI, R_1676_/R_1933_, R_750_/R_550_, and R_950_/R_750_.

## 1. Introduction

Anthropogenic activities can result in the release of a wide array of contaminants, particularly metals, to the environment. Responsible environmental stewardship involves the management and remediation of such releases. Numerous remediation strategies exist, depending on the circumstance, but the technique considered here is reflectance spectroscopy. Reflectance spectroscopy has potential for use as a cost-effective, non-destructive analytical technique for detecting and assessing plant stress, specifically metal stress [1,2,3].

Chemometric mathematical methods are often used to analyze reflectance spectra when extensive samples are available; a few hundred are required to develop a robust model. For smaller sample sizes, as in this study, vegetation indices (VI; mathematical combinations of different reflectance spectral bands) can be used to provide rapid and convenient semi-analytical measures of vegetation activity, which in turn can provide indication of plant health [4].

The ability to detect metal exposure in plants is particularly relevant when employing phytoremediation strategies. Phytoremediation is defined, for our purposes, as the use of green plants for environmental clean-up, or the use of plants to remove or neutralize pollutants in the biosphere [5]. Specific applications for reflectance spectroscopy as a tool complimentary to phytoremediation could include: (1) early recognition of contaminated areas through identification of plant stress indicative of metal exposure, (2) surveillance of sites with existing contamination or sites with the potential to become contaminated, (3) assessment of phytoremediation efforts, (4) assessment of risk to human health and the environment through coarse quantification of contamination, etc.

The contaminant of concern in this study is lithium. Lithium (Li) is widely used in the USA, which is the leading producer and consumer of Li materials, finding utility in ceramics and glass, aluminum production, the medical industry, certain batteries and greases, nuclear reactor coolant, radiation dosimeters, and historically, in nuclear weapon development [6,7,8,9]. Although Li is not a radioactive concern, it is an anthropogenic contaminant related to the nuclear fuel cycle and to legacy waste and contamination from nuclear weapons development [8,10,11]. For example, historical waste-disposal activities at the Department of Energy’s Oak Ridge Y-12 Plant resulted in the release of Li to the groundwater [8]. Numerous studies have shown that there are shifts in plant reflectance spectra due to metal stress (or simulated metal stress) [2,12,13,14,15,16,17,18,19,20,21,22,23,24] but to the authors’ knowledge none have considered response to Li exposure.

To further investigate the use of reflectance spectroscopy as a useful method for assessing metal stress in plants, reflectance spectra for four species of plants were collected every week for three weeks using an ASD FieldSpec Pro spectroradiometer with both a contact probe and a field of view (FOV) probe. These plants were treated twice weekly in a laboratory setting with 0 mM (control) or 15 mM of lithium chloride (LiCl) solution and harvested weekly, immediately after spectra collection, for assessment of physical endpoints such as relative water content and chlorophyll content. The specific objectives of this exploratory study are to: (1) identify changes in plant status due to Li exposure in multiple plant species and (2) determine if reflectance is useful in identifying these changes through the utilization of both previously defined and newly determined vegetation indices.

### 1.1. Plant Species Considered

The species considered were *Arabidopsis thaliana*, *Helianthus annuus* (sunflower), *Brassica napus* (rape), and *Zea mays* (corn). *A. thaliana* is a member of the mustard family that is closely related to various crop plants. It has been the subject of intense study over the past several decades and is considered a model organism and ideal for use in the laboratory setting for biological research [25]. Several species of the *Brassica* family, which are vegetable and oilseed crops, have been identified as metal accumulators and are considered potential phytoremediation candidates [26,27,28]. *H. annuus* is an ornamental flower as well as an important environmental crop, and it has been shown to be an effective phytoremediation crop [29,30,31]. *Z. mays* has also been shown to have metal phytoremediation potential [31]. It is the major feed grain (>95%) in the United States and is also processed into a broad assortment of food stuffs, from cereals to sweeteners. Additionally, *Z. mays* has industrial utility as a component in the fabrication of fuel ethanol. The United States is currently the world’s largest producer and exporter of corn [32]. Several studies have considered the reflectance spectra of corn and sunflower, from assessing pigment concentrations to nutrient/water status and photosynthetic efficiency at both leaf and canopy scales [33,34,35,36,37,38,39,40,41,42,43,44,45].

### 1.2. Lithium in Plants

Lithium is the lightest metal, although as it is highly reactive, does not occur naturally in its elemental form. It occurs in various minerals and salts, and typically enters the environment through weathering processes. Once in the environment, Li is easily transported to above-ground plant biomass as it shares the potassium (K^+^) transport carrier; all plants will take up Li [7,8,9]. 

Lithium uptake, tolerance, and toxicity symptoms are all species specific, although stimulatory effects are commonly seen at low levels of Li [7,9,46,47]. Although symptoms of lithium toxicity are not distinct between species, general symptoms include chlorosis, necrotic spot development, leaf curling, and reduced biomass, all similar symptoms as exhibited in pathogen defense [48,49]. There is preferential uptake of Li in older leaves, so effects will be less pronounced on new growth [49,50,51]. LiCl has been used previously to consider Li uptake and stress, but although chlorine (Cl) is an essential micronutrient for higher plants, at high plant tissue concentrations Cl as the chloride ion (Cl^−^) can be toxic [7,26,52,53]. However, the concentrations of LiCl used here are considered below levels of which Cl might be toxic; contribution of Cl to the effects in this case are considered negligible [52,53].

Several studies have considered Li toxicity in plants [7,26,47,48,49,50,51,54,55,56,57,58] although the mechanism is not fully understood; plants have each type of enzyme shown in animals and yeast, respectively, to be Li sensitive, namely inositol monophosphatase and HAL2 nucleotidase. It is fairly unknown which of these two enzymes is the major target of Li action [54]. Generally, at high concentrations, Li increases the production of ethylene, which is known to inhibit plant growth. The mechanism is not wholly understood, although the “inositol depletion hypothesis” is generally accepted. This theory holds that Li^+^ inhibits inositol monophosphatase, which ultimately triggers ACC (aminocyclopropane carboxylic acid) synthase, resulting in an increase in ethylene biosynthesis [7,49,54,55]. There is also evidence to suggest that Li toxicity can phenotypically resemble magnesium (Mg) deficiency and that Li^+^ can bind to proteins (such as chlorophylls) that normally bind with Mg^2+^ [56].

## 2. Materials and Methods

### 2.1. Plant Growth and Treatment

The soil mix used was a 4:1 mixture of PGX (Promix PGX, Premier Horticulture Inc., Quakertown, PA, USA) and perlite (Hoffman Horticultural Perlite, Good Earth, Lancaster, NY, USA). Soil was mixed and placed in square plastic grow pots (10.8 × 10.8 × 12.7 cm) with perforated bottoms to allow water seepage; soil was well-hydrated prior to sowing seeds. Nutrient solution used to water and treat plants was made with DI water, 1/32 strength Murashige and Skoog basal medium (137.5 mg L^−1^) (cat. no. M5519, Sigma-Aldrich, St. Louis, MO, USA), and 250 mg L^−1^ MES hydrate (cat. no. M2933, Sigma-Aldrich), using KOH to pH balance to 5.7. *A. thaliana* seeds (WT-02-41-01 Columbia [alias Col-0] Wildtype, LEHLE Seeds, Round Rock, TX, USA) were soaked in nutrient solution, exposed to red light for 30 min to synchronize germination, and pipetted onto the soil pots. *Z. mays* seeds (Burpee Sweet Corn Bi-Licious Hybrid, Burpee Garden Products Co, Warminster, PA, USA) *H. annuus* seeds (Snow Country Black Oil Sunflowers, Ridley Inc., Mankato, MN, USA), and *B. napus* seeds (Winfred Brassica Rape, outsidepride.com, lot: M31-9-2WIN, Independence, OR, USA) were planted in the soil pots by hand. Following the sowing of the seeds, the 1/32 nutrient solution was further diluted to 1/64 strength for subsequent treatments of all plant species.

After planting, arbitrary sets of 6 pots each were transferred to plastic tubs (40 × 31.75 × 15.24 cm) in 3 cm distilled water. Tubs were placed in rows of up to four on growth shelves, 42 cm beneath growth lights. Plants were on a 9 h light: 15 h dark cycle under ambient laboratory environmental conditions. At the seedling stage, plants were culled and/or redistributed to ensure an appropriate number of plants in each pot, based on the appearance of health: *A. thaliana* were culled to three seedlings per pot, *B. napus* were culled to 10 plants per pot, *Z. mays* were culled to three plants per pot, and *H. annuus* were culled to one plant per pot. Immediately prior to LiCl treatment, pots were randomly rearranged between tubs (six pots per tub, no longer in DI water) such that each tub, now serving as a treatment group, had similar size and quality plants. Each experiment had three treatment group tubs and three control tubs for a total of 36 samples (i.e., plants) per experiment. Each experiment was conducted twice such that there was a total of 72 plant samples per species, or 288 total samples. 

Once pots were arranged for treatment, spike solution was evenly applied to the top of each pot as 100 mL (25 mL delivered to each quadrant) of 15 mM LiCl in 1/64 strength nutrient solution twice weekly, with control plants receiving 100 mL 1/64 nutrient solution only. Two pots were randomly selected from each treatment group for weekly spectra collection and harvest. After each application of nutrient solution, the plants were rotated within the tubs and the tubs were rearranged among the growth shelves to account for potential variation in lighting or other environmental conditions.

### 2.2. Equipment Setup and Spectra Collection

Reflectance spectra were collected using a FieldSpec Pro (FSP 350-2500P; Analytical Spectral Devices (ASD), Boulder, CO, USA) which is a full range (350 nm to 2500 nm) portable spectroradiometer (with sampling intervals/spectral resolutions of 1.4 nm/3 nm and 2 nm/10 nm for 350 to 1000 nm and 1000 to 2500 nm respectively) [59]. Contact probe (CP) spectra were collected using a leaf clip attachment on individual leaves. The CP provides light (3.825 V, 4.05 W low intensity bulb) and collects reflectance spectra. The leaf clip attachment has both a white (for white reference) and black (to minimize back scatter) background. Multiple reflectance readings were taken on the leaves of each species of plant to obtain an overall representation of reflectance. 

FOV spectra were collected using an 8° probe (i.e., a viewing angle of 8°). Incident light was provided by two halogen lamps (Pro Lamp, 14.5 V, 50 W, P/N 145378, ASD) angled at 30 degrees from horizontal. The lights were 180° apart at 30.5 cm from the center of pot on the horizontal and 76.2 cm above the table surface. The fore optics probe was centered between the lights at 66.7 cm above the plane of the pot surface, resulting in a spot size diameter of 9.32 cm. Reflective surfaces were covered with light-absorbent material to minimize noise and thus variability in spectra, and dark room conditions were approximated by surrounding the lights and fore optics with a black felt canopy. Tripod surfaces were also wrapped in black felt and the table surface was lined with light-absorbent black rubber. The white reference was a calibrated Spectralon (25.4 × 25.4 cm, LabSphere, North Sutton, NH, USA) panel of 99% reflectance that was elevated to a height equivalent to a grow pot. Four spectra, each collected at a different arbitrary rotation of the pot, were acquired and then averaged to get an overall assessment of the reflectance of the sample. FOV spectra were always acquired prior to CP because it is possible for the CP to injure the plant and therefore affect subsequent FOV readings.

### 2.3. Collection of Physical Measures

As metal stress is known to mimic drought stress [3], plants were harvested after spectra collection each week to determine chlorophyll content and relative water content. The concentrations of chlorophyll *a* (Chl *a*) and chlorophyll *b* (Chl *b*) were determined for each replicate (i.e., pot) [26,60,61]. Four circular leaf subsamples were collected from representative leaves of the plants in a pot using a #3 cork borer (Fisher Scientific, Pittsburgh, PA, USA). Leaf samples were stored in the dark at 4 °C in capped 20 mL vials (KG-33 borosilicate glass; Kimble Chase, Vineland, NJ, USA) containing 2 mL 100% ethanol for three days before absorbance (*A*) at 665 nm, 649 nm, 629 nm, and 696 nm, with an offset at 750 nm, was determined for 1.5 mL subsamples for each vial using a NanoDrop 2000c UV-Vis spectrophotometer (Thermo Scientific, Wilmington, DE, USA). Disposable methacrylate cuvettes with transmission from 300 to 800 nm >80% were used with the 1.5 mL subsample (Cole Palmer, Vernon Hills, IL, USA). Chlorophyll content was determined using appropriate, previously published [61] equations where $A_{x}$ is absorbance at *x* nm:

(1) $$\mathrm{Chl}\;a\;\left(\mu g/\mathrm{mL}\right)=-5.2007\cdot A_{649}+13.5275\cdot A_{665},$$

(2) $$\mathrm{Chl}\;b\;\left(\mu g/\mathrm{mL}\right)=22.4327\cdot A_{649}-7.0741\cdot A_{665}.$$

After leaves were sampled for chlorophyll content, additional sufficient leaves were removed to obtain between 1000 and 2000 mg of fresh mass for each replicate (i.e., pot) to determine relative water content. Samples were placed in weigh boats, fresh mass was obtained, samples were dried in an oven to a constant mass, and dry mass was obtained. A sample’s relative water content (RWC) was then expressed as Equation (3):

(3) $$\mathrm{RWC}=1-\frac{\mathrm{dry}\;\mathrm{mass}\;}{\mathrm{fresh}\;\mathrm{mass}}$$

Lithium uptake has been shown to have a stimulatory effect at low levels, and potentially cause necrosis at high levels depending on the plant species [47,48], so remaining plant material from above was similarly retained, dried, and weighed to determine each sample’s overall dry biomass. Note that dry biomass was determined for all experiments except the first *A. thaliana* experiment, as these samples were initially used elsewhere for teaching purposes. 

A visual assessment of the proportion of a pot covered by plant material was performed by overlaying a 6 × 6 (18 mm × 18 mm) grid on top-down photos of each group of plants at each of three timepoints, forming 36 squares with 49 evenly-spaced points (grid intersections). Photographs were taken immediately prior to spectra collection, directly above each six-pot treatment group and six-pot control group in the same manner each week. However, to account for any potential change in magnification or alignment, gridlines were laid based on pot dimensions, which were definitively consistent. Using the grid intersections, an additional endpoint, Coarse Leaf Area Index (CLAI), was defined as:

(4) $$\mathrm{CLAI}=\frac{N_{\mathrm{leaf}\;}}{N_{\mathrm{total}}}$$

where *N*_total_ is the total number of points in the grid and *N*_leaf_ is the number of points on leaf material. CLAI provides an approximate indication of how much of the pot surface is covered by plant material. Finally, heights of plants above the top of the pot were measured. The only plants with noticeable variation in heights were *H. annuus* and thus are the only results reported for plant height.

### 2.4. Data Analysis

Initially, fifteen VI were considered for applicable spectra acquisition technique(s) (i.e., FOV and/or CP), including indices from the literature [e.g., normalized difference vegetation index (NDVI), photochemical reflective index (PRI), normalized phaeophytinization index (NPQI), structural independent pigment index (SIPI), pigment specific normalized difference (PSND), normalized difference index (NDI), water index (WI), yellowness index (YI)] [15,19,39,62,63,64,65,66,67,68,69,70,71]. Six VI (R_1390_/R_1454_, PRI, SIPI, NDI, R_1110_/R_810_, and R_725_/R_675_) were removed from consideration as they were highly colinear with other VI. Pearson correlation coefficients (PCC) were utilized to determine collinearity, and VI were considered for exclusion if the absolute value of the PCC between two (or more) was greater than 0.75 for CP and 0.50 for FOV. Preference was given to VI previously associated with metal exposure. 

For each species at each view, a mixed-effects model analysis was conducted for each vegetation index and each plant endpoint (RWC, Chl *a + b*, dry biomass, CLAI, and/or height) to consider the fixed effects of week (1, 2, 3), treatment group (control or 15 mM LiCl treatments), and week-by-treatment interaction with a random effect for sampling unit. The Kenward-Rogers approximation of degrees of freedom was used to account for variation among the week-by-treatment combinations. When a treatment effect or week-by-treatment interaction was significant for a plant stress endpoint (RWC, Chl *a + b*, biomass, CLAI, and/or height), subsequent linear mixed-effects analyses were conducted to consider the endpoint measure as a dependent variable with vegetation indices as predictors that also had significant treatment or week-by-treatment interaction effects from the mixed-effects model analysis as predictors. SAS Software v. 9.4 (SAS Institute Inc., Cary, NC, USA) was used for all statistical analyses and a significance level of 0.05 was used for all tests of significance.

## 3. Results

### 3.1. Spectra

**Figure 1:**
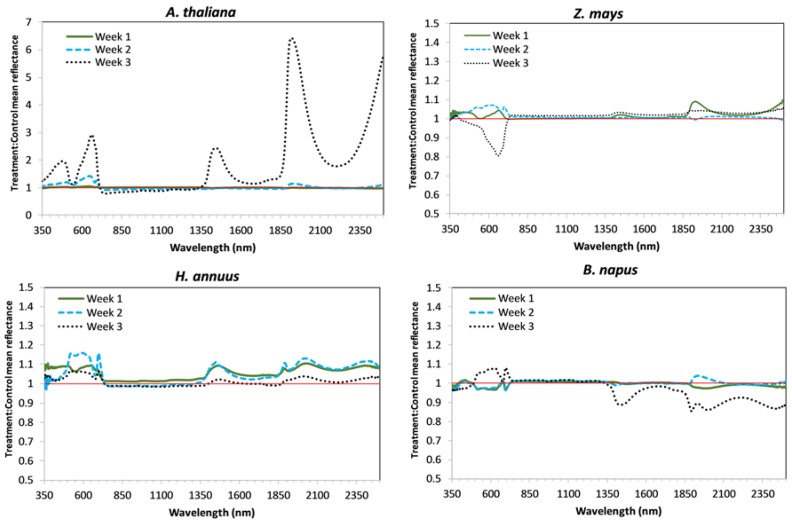
Relative reflectance of treatment to control for each of four species as acquired by CP.

**Figure 2:**
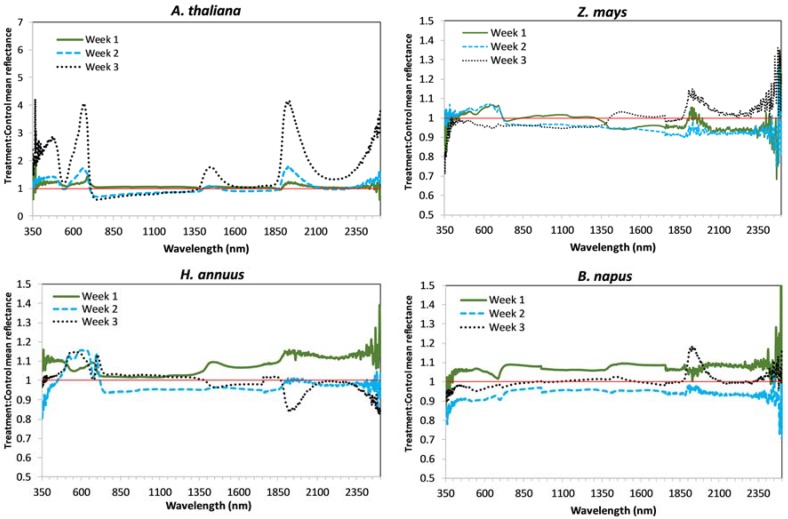
Relative reflectance of treatment to control for each of four species as acquired by FOV.

**Figure A1:**
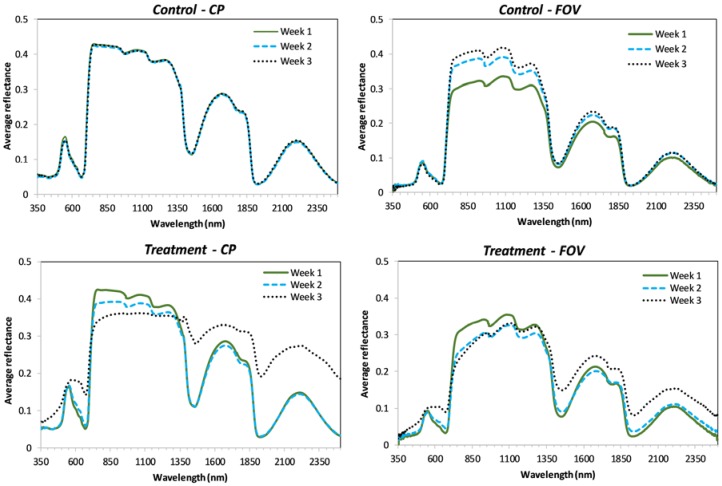
Control and treatment spectra for *A. thaliana* as acquired by CP and FOV.

**Figure A2:**
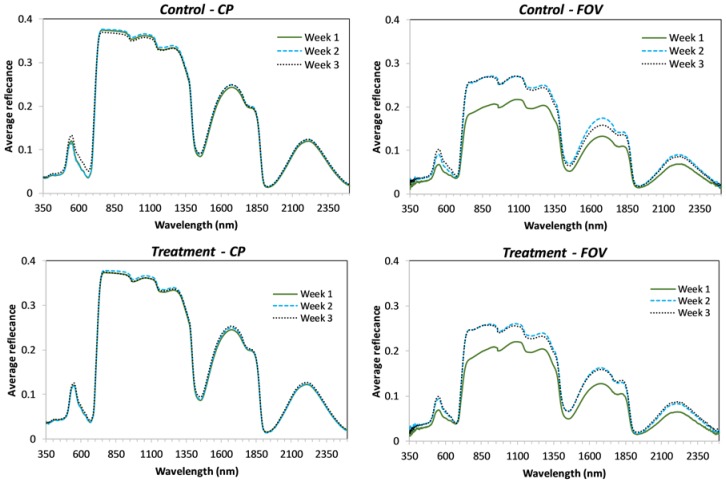
Control and treatment spectra for *Z. mays* as acquired by CP and FOV.

**Figure A3:**
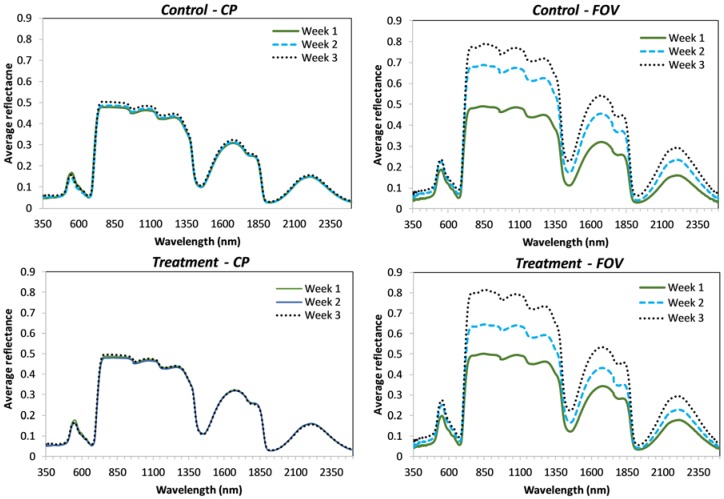
Control and treatment spectra for *H. annuus* as acquired by CP and FOV.

**Figure A4:**
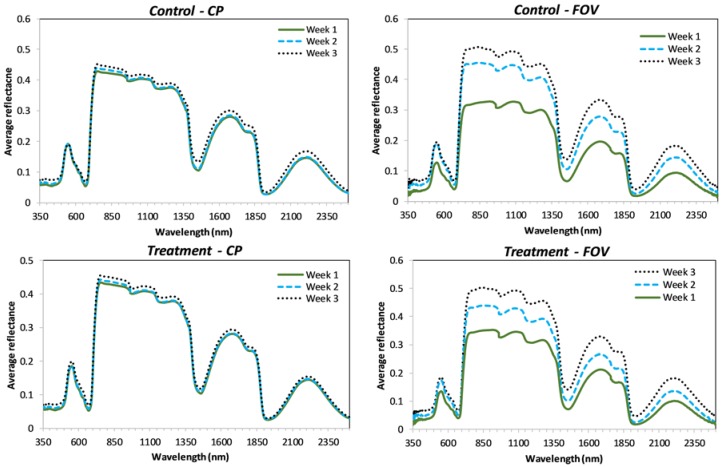
Control and treatment spectra for *B. napus* as acquired by CP and FOV.

Average relative reflectance spectra of treatment compared to control are shown by week and technique (CP or FOV) for each species in Figure 1 (CP) and Figure 2 (FOV). Average reflectance spectra for each experimental group (i.e., control and treatment) are shown in Appendix A (Figure A1, Figure A2, Figure A3 and Figure A4). Average reflectance data (from which the figures were developed) is included in the supplemental online material. Notice that the vertical axes in Figure 1 and Figure 2 are consistent between *Z. mays*, *H. annuus*, and *B. napus*, but needed an extended range for *A. thaliana*.

### 3.2. Phenotypic Observations

**Figure 3:**
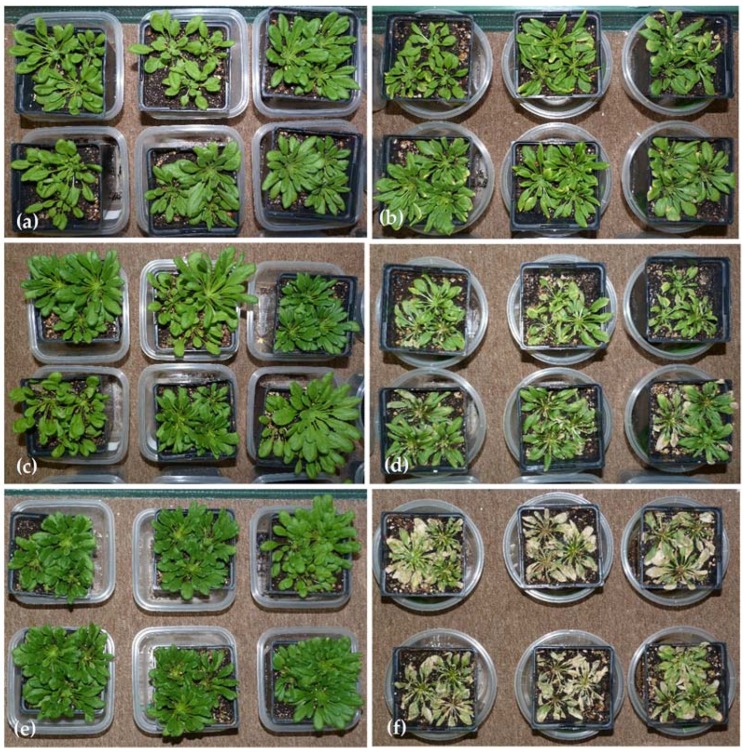
Weeks 1, 2, and 3 of the first *A. thaliana* experiment; response was similar in the second experiment. (**a**) Week 1 control plants; (**b**) Week 1 treatment plants which exhibit slight chlorosis along the edges of older leaves; (**c**) Week 2 control plants; (**d**) Week 2 treatment plants which exhibit chlorosis, necrosis, and decreased biomass; (**e**) Week 3 control plants; (**f**) Week 3 plants which exhibit significant necrosis.

*A. thaliana* had visible symptoms of Li exposure starting in week 1, from a slight yellowing (chlorosis) at the leaf tips in week 1 to significant necrosis in week 3 (Figure 3). 

**Figure 4:**
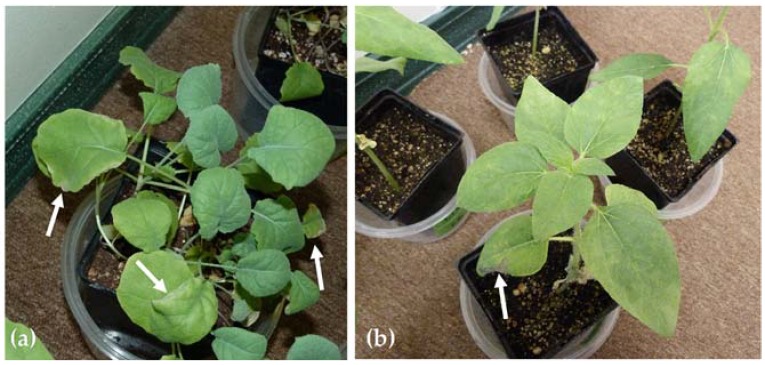
Example (**a**) *B. napus* and (**b**) *H. annuus* plants at week 3. Arrows in (**a**) are pointing out areas of chlorosis around leaf edges. The arrow in (**b**) is pointing out a necrotic spot.

*B. napus* plants showed some symptoms of toxicity around leaf edges in weeks 2 and 3 (Figure 4a). A few *H. annuus* plants began exhibiting slight symptoms of lithium toxicity between weeks 2 and 3, as slightly mottled leaves and occasional necrotic spots (Figure 4b). *Z. mays* showed no symptoms of toxicity. Observed symptoms are typical of lithium toxicity; different species of plants are known to have varying tolerances to lithium exposure [48,72].

### 3.3. Endpoints

**Figure 5:**
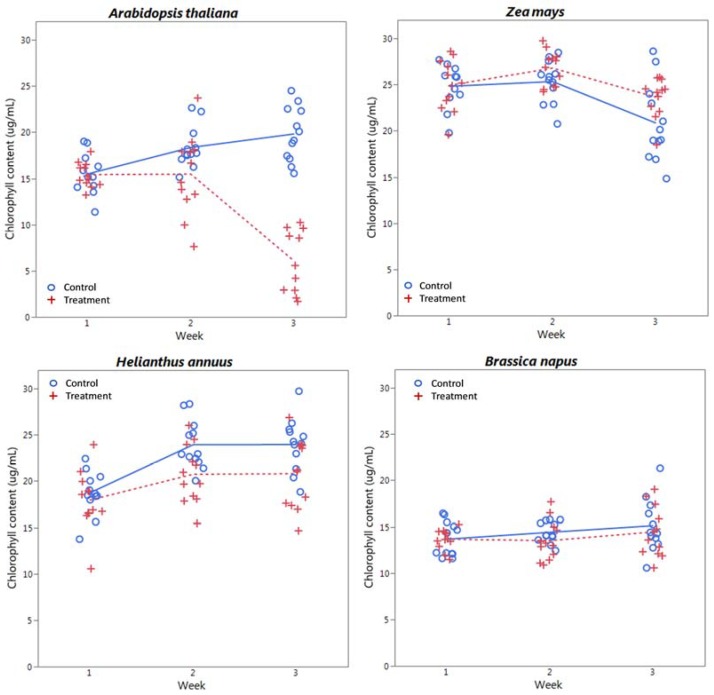
Chlorophyll content (Chl *a + b*, µg/mL) by week and treatment level for each species considered. Lines (solid = control, dashed = treatment) indicate mean values at each week.

**Figure 6:**
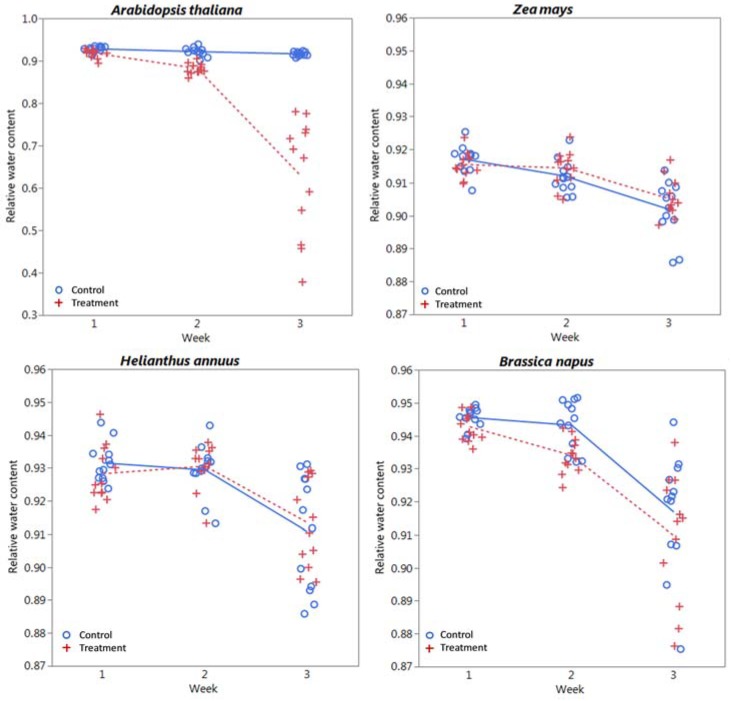
Relative water content (RWC) by week and treatment level for each species considered. Lines (solid = control, dashed = treatment) indicate mean values at each week.

**Figure 7:**
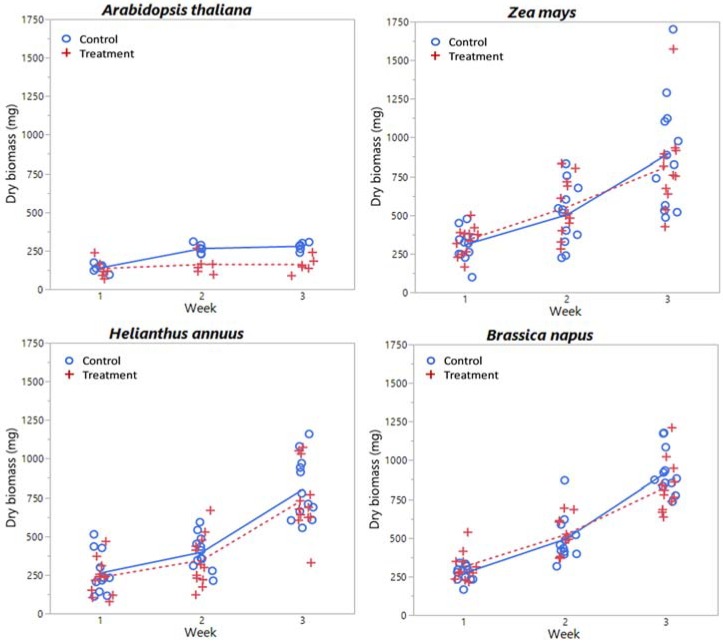
Dry biomass (mg) by week and treatment level for each species considered. Lines (solid = control, dashed = treatment) indicate mean values at each week.

**Figure 8:**
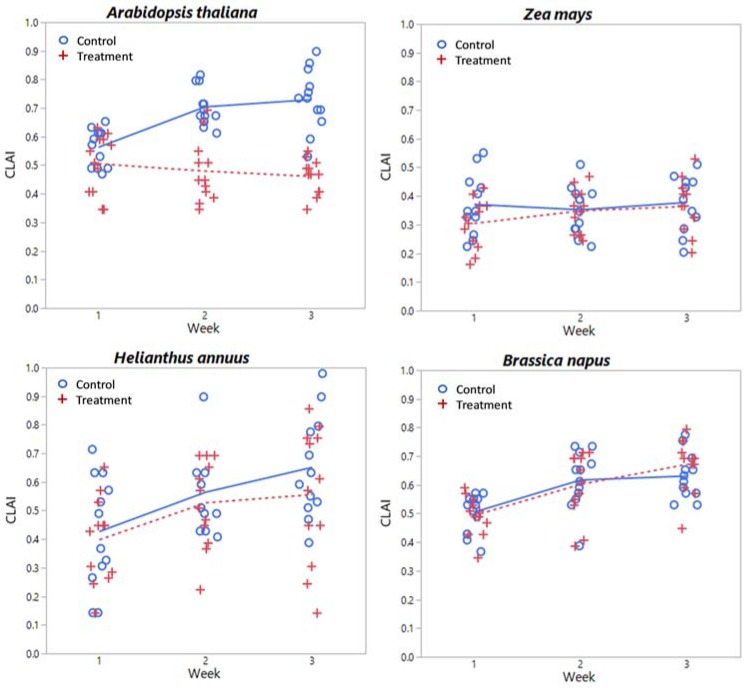
CLAI by week and treatment level for each species considered. Lines (solid = control, dashed = treatment) indicate mean values at each week.

**Figure 9:**
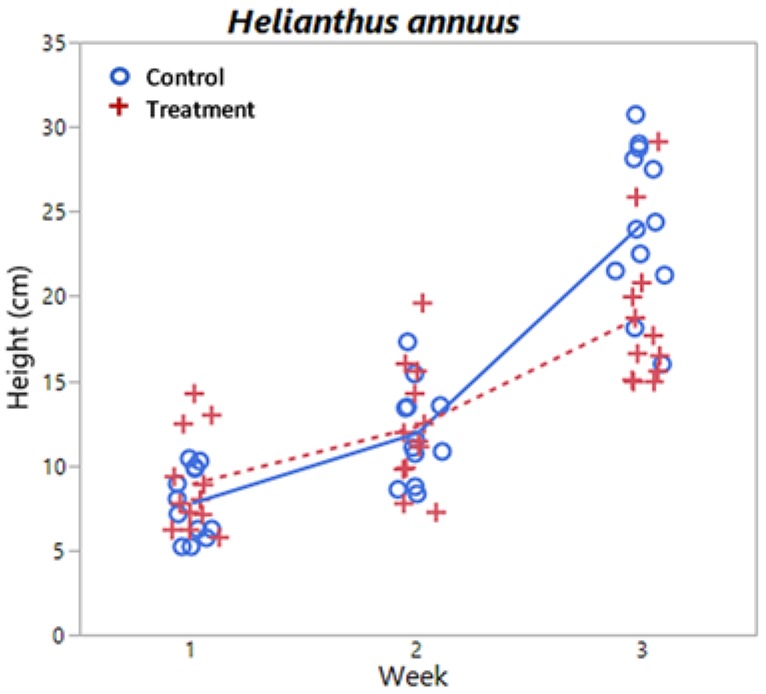
Height by week and treatment level for *H. annuus*. Lines (solid = control, dashed = treatment) indicate mean values at each week.

Plots of chlorophyll content, relative water content, dry biomass, and CLAI by treatment level and time are shown in Figure 5, Figure 6, Figure 7 and Figure 8 respectively for each species. A plot of *H. annuus* height is shown in Figure 9. Summary statistics for these endpoints are provided in Appendix B. Notice in Figure 6 (RWC) that the vertical axis is different for *A. thaliana* compared to the other species as the treatment plants had a substantially greater range of values. Other figures depicting endpoint results have consistent scales on the vertical axes.

Results (*p*-values) of the statistical analysis for relative water content, chlorophyll content, dry biomass, CLAI, and height are shown in Table 1. *F*(*n*,*d*) in table headers indicates the degrees of freedom with which the *F* test statistic and associated *p*-value were calculated, where *n* is numerator degrees of freedom, and *d* denominator degrees of freedom. Note that denominator degrees of freedom vary due to differing numbers of observations and Kenward-Rogers degrees of freedom approximation. There are no significant time, treatment, or time by treatment interaction effects for *B. napus* chlorophyll content, nor for *Z. mays* CLAI. All other endpoints had significant differences in time for each species. There was a significant treatment effect on Chl *a + b* and a significant time by treatment interaction effect on height for *H. annuus*. 

### 3.4. Vegetation Indices and Overall Response

Detailed results (*p*-values) from the initial mixed-effects analysis of the vegetation indices are contained in Appendix C. Significant *p*-values (*p* < 0.05; bolded) indicate a statistically significant difference between the treatment, week, or treatment-by-week interaction outcome means. As above, denominator degrees of freedom vary. From these values we see that methods of spectra acquisition as well as the results between species are generally varied. However, this is not wholly unexpected as four fairly different species were considered; these species vary in leaf size, height, structure, etc.

Nearly all *A. thaliana* VIs had significant treatment, time, and interaction effects between treatment and time, for both spectra acquisition techniques (CP and FOV). For other species, however, results differ between VI, spectra acquisition techniques, endpoints, and time/treatment. Every species exhibited significant time-dependent differences in VI. Corn only had significant treatment effects for YI acquired by FOV. *H. annuus* had significant treatment or interaction differences in PSND, YI, R_750_/R_550_, and R_1636_/R_1933_. *B. napus* had significant treatment or interaction differences in WI, YI, R_750/R550_, and R_1636/1933_. However, only *A. thaliana* and *H. annuus* had significant treatment effects with respect to measured endpoints (RWC, Chl *a + b*, biomass, CLAI, and/or height) (Table 1), so only these species were considered in secondary (i.e., follow-up) analysis to determine which VIs were significant predictors of these endpoints. Results of the corresponding mixed effects model are shown in Table 2 below. The only index with no predictive power was WI; all other VI were significant predictors of at least two endpoints. Interestingly, R_750_/R_550_ was a significant predictor in at least one view (CP or FOV) of all endpoints considered, as further discussed below. This index is therefore deemed the most promising of those considered for prediction of lithium exposure.

## 4. Discussion

In this study, four species of plants were treated with lithium chloride over three weeks to discern if symptoms of the exposure could be identified from their reflectance spectra through the use of VI. Plants were harvested each week, with reflectance spectra collected at the whole plant and leaf scales. Various physical endpoints were measured immediately after spectra acquisition. Controls were maintained in parallel with the treatment plants at the same sample size, as matching controls are important to be able to discern if temporal changes in reflectance result from contaminant exposure or from the natural growth pattern or inherent biological variability of the plants [17,22,69].

The progressive Li exposure had slightly different effects, as well as severity of effects depending on the species. All species considered exhibited significant changes in time, but only physical measures of *H. annuus* and *A. thaliana* exhibited significant treatment effects. Although *Z. mays* did not demonstrate any obvious symptoms of Li toxicity, *B. napus* did show slight symptoms of toxicity around the leaf edges starting in week 1. Uptake of Li is expected to be higher in dicots (e.g., *A. thaliana*, *H. annuus*, and *B. napus*) than in monocots (e.g., *Z. mays*) [7], which might explain why no symptoms were seen in *Z. mays*. Similarly, the low biomass of *A. thaliana* as compared to plants with much higher biomass for an equivalent soil concentration may have led to the more severe toxicity symptoms.

Observed symptoms in *A. thaliana*, *H. annuus*, and *B. napus* were consistent with Li toxicity, which in turn are similar to those resulting from Mg deficiency [56]. Mg is essential for photosynthesis as it is the central element of the chlorophyll molecule [73]. It is possible that the competitive binding of Li results in Mg deficiency, exacerbating the toxicity symptoms. Of the initial 15 VI considered, seven were found to be significant predictors of these treatment-dependent endpoints. Comparisons between species and the associated relationships with the acquired spectra and calculated VIs are discussed below.

### 4.1. Chlorophyll Content

Excess metal exposure negatively affects photosynthetic processes and typically induces a general stress reaction in plants [74]. Photosynthetic pigments typically decrease with metal exposure, which has obvious consequences for photosynthesis and plant growth. Inhibition of photosynthesis is one effect that most metals have in common when present at toxic concentrations; reduction in photosynthetic efficiency will be seen as an increase in reflectance in the visible range, as less light is being utilized for photosynthesis and chlorophyll production [3].

*A. thaliana* and *H. annuus* showed significant changes in chlorophyll content by treatment level, which corresponds to the increase in reflectance in the visible region (see Figure 1 and Figure 2; notice differences in *H. annuus* are subtle compared to *A. thaliana*). Interestingly, for *H. annuus,* chlorophyll content increased from week 1 to week 2, but remained essentially constant from week 2 to week 3, whereas *Z. mays* chlorophyll content remained the same from week 1 to week 2 and decreased from week 2 to week 3 (Figure 5). Also, although not statistically significant, chlorophyll content in treatment plants appears to increase slightly above that in control plants in *Z. mays* in week 3 (Figure 5), which corresponds to the increase in absorbance (and thus decrease in reflectance) seen in the *Z. mays* spectra in Figure 1 and Figure 2. There were no significant differences in *B. napus* chlorophyll content by week or treatment (Table 1), and *B. napus* also generally had lower chlorophyll content than the other species (Figure 5 and Table A1); *Z. mays* and *H. annuus* had the highest chlorophyll contents.

The vegetation indices that proved to be significant predictors of chlorophyll content for *H. annuus* and *A. thaliana* were PSND, YI, R_750_/R_550,_ and R_1636_/R_1933_. PSND, which is determined by the normalized difference (R_800_ − R_680_)/(R_800_ + R_680_), has been shown to be correlated with pigment concentration per unit area [70]. Here, PSND was found to be a significant predictor of chlorophyll content only for spectra acquired by CP, not FOV (Table 2). However, for *A. thaliana*, PSND as determined using FOV was a significant predictor of CLAI. Taken together, this is consistent with previous findings. Note that CLAI did not have significant treatment differences for *H. annuus*, and PSND also did not have significant treatment differences for *H. annuus* spectra acquired via FOV. Similarly, R_1636_/R_1933_, which has been previously suggested to be associated with cesium (Cs, also an alkali metal) exposure in *A. thaliana* [69], was also a significant predictor of chlorophyll content when assessed using CP spectra, and CLAI when assessed utilizing FOV spectra.

YI is an approximated second derivative of the spectra at about 600 nm and is intended to provide indication of chlorosis, i.e., leaf yellowing [67]. In this study, YI was found to be a significant predictor of chlorophyll content with FOV spectra for *H. annuus* and with CP spectra for *A. thaliana*. As the YI was originally developed at the CP scale, it is likely that soil background will influence this VI, particularly in the instances like that of *A. thaliana* week 3 treatment plants where much of the soil is exposed to the fore optics. *H. annuus* leaves are large and broad and, being closer to the fore optics than *A. thaliana* plants (and much closer than the soil), leaf reflectance dominates the spectra. Also, FOV may potentially capture the mottled nature of the yellowing of *H. annuus* leaves better than CP. R_750_/R_550_ was originally developed for remote sensing of chlorophyll [68,71] but has also been found to be correlated with metal content [15]. Here, R_750_/R_550_ was found to be a significant predictor of chlorophyll content at both CP and FOV scales for both species considered. 

### 4.2. Water Content

Metals can disrupt the plant-water balance [3], which can be seen as an increase in reflectance in the mid-infrared region, as water absorbs fairly strongly at 1450 nm, 1940 nm, and 2500 nm, with slight increase in the near infrared region, associated with slight water absorption [75,76]. *A. thaliana* was the only species that exhibited significant treatment effects associated with RWC (Table 1), although all species exhibited significant temporal differences, with RWC decreasing in time (Figure 6). The difference in treatment versus control reflectance for *A. thaliana* is clear in Figure 1 and Figure 2 as spikes in relative reflectance at the aforementioned water absorption wavelengths that get larger each week. Although difficult to see in the relative spectra, temporal differences in the remaining species can be seen in the average (i.e., raw) reflectance spectra as an increase in reflectance at these same wavelengths in both treatment and control plants (Figure A2, Figure A3 and Figure A4). Differences for each species except corn are greater between weeks 2 and 3 than between weeks 1 and 2, although the overall range of RWC is narrow for control plants, varying by only a few percent. It is interesting to note that the 1940 nm band in Figure 2 appears to give a qualitatively more consistent relative response between weeks than the other water bands. Although not statistically significant, *B. napus* RWC of treatment plants appears to decrease in time more than the control plants. This species, along with *H. annuus* had increasing variability (i.e., standard deviation) in RWC with time (Table A1), which explains why the apparent difference is not statistically significant. 

The vegetation indices that proved to be significant predictors of RWC for *A. thaliana* were R_750_/R_550_ with FOV, R_950_/R_750_ with CP, and (R_950_ − R_750_)/(R_950_ + R_750_) with CP. The ratio (R_950_ − R_750_)/(R_950_ + R_750_) was considered because normalized differences are frequently utilized to improve upon a simple ratio by accounting for background reflectance [71]. In this instance, the normalized ratio has similar significance to its corresponding simple ratio, R_950_/R_750_, providing good indication that background reflectance is not a confounding factor. Similar to R_1636_/R_1933_ discussed above, R_950_/R_750_ was previously found to be associated with Cs exposure in *A. thaliana*, being correlated with RWC and Chl *a + b* at the CP scale and CLAI at the FOV scale in Cs contaminated plants [69]. Here, R_950_/R_750_ was not found to be significant for Chl *a + b* or CLAI (although p = 0.0661 [CP] and p = 0.0663 [FOV], which would be significant at a 90% confidence level), but it is found to be significant for RWC with CP. Also consistent with this study is that the Cs study also found R_750_/R_550_ at the FOV scale to be a significant predictor of RWC. This suggests that, as mentioned above, R_750_/R_550_ along with R_950_/R_750_, may be good predictors of plants exposed to alkali metals.

### 4.3. Remaining Endpoints: Size and Shape

Although there are no significant treatment effects, *Z. mays*, *B. napus*, and *H. annuus* share similar magnitudes of and temporal changes in dry biomass (Figure 7). By week 3, plants have more than doubled their week 1 biomass, and *B. napus* has the highest average mass followed by *Z. mays*, then *H. annuus*, although *Z. mays* has the largest variation in biomass; the maximum *Z. mays* biomass was about 1700 mg, followed by a maximum of about 1200 mg for both *H. annuus* and *B. napus*. The temporal increase in FOV acquired reflectance for these species (for both treatment and control groups) in the near-IR is partially due to the increase in biomass of the plants (Figure A2, Figure A3 and Figure A4). For *H. annuus*, not only do the plants get taller (Figure 7), and therefore become closer to the fore optics, but there is also an increase in number and size of leaves. *H. annuus* leaves are broad and grow parallel to horizontal, resulting in a large reflective surface perpendicular to the fore optics. There will be an increase in light scattering within a plant containing a greater proportion of cell surfaces exposed to intercellular air space, due to different indices of refraction of these materials. As near infrared light is not used for photosynthesis, increasing light scatter in the infrared region means less transmission of light through the plant and more reflection back to the fore optics. Differences in plant structure will therefore affect reflectance of light in the near-IR; larger leaf areas will result in higher reflectance, whereas cell degradation or reduction in leaf thickness will result in lower reflectance, as there will be less light scattering within the plant leaf [77].

Although the mean CLAI for *H. annuus* is comparable to that for *B. napus* (~50%–60% coverage), the variability is much greater with some plants at almost 100% coverage by week 3. Also, *B. napus* grew less than 10 cm above the top of the pot so are much further away from the fore optics than *H. annuus*. Although there were no differences in *Z. mays* heights between groups (details not shown), plants grew to ~45 cm in week 3, about double the *H. annuus* control plant mean (Table A2). However, the leaves are narrow and grow at an angle vice horizontal, meaning there is less leaf surface directly exposed to the fore optics compared to *H. annuus*. In fact, there are no significant differences in time or treatment for Z. mays CLAI, which remains essentially constant at ~35% coverage. These points together can be seen as differences between species in the magnitude of reflectance in the near-IR of FOV spectra in Figure A2, Figure A3 and Figure A4. For *Z. mays*, reflectance in the near-IR varies between ~0.18 and 0.25, for *B. napus* between ~0.32 to 0.50, and for *H. annuus* between ~0.48 to 0.80 from weeks 1 to 3.

Contrasting with the other three species, *A. thaliana* had significant treatment and temporal differences in biomass. *A. thaliana* plants also had much less biomass than the other species considered, as they are small, squatty plants prior to flowering. The dry biomass maximum for *A. thaliana* was less than 300 mg. Field-of-view control reflectance for *A. thaliana* increased in the near-IR each week (Figure A1), corresponding to the increase in biomass of the control plants. Treatment reflectance in the near-IR was lower than the control reflectance for both the CP and FOV in weeks 2 and 3 (Figure 1 and Figure 2); the lower CP reflectance indicates a difference in leaf structure. Lower FOV reflectance indicates a lower biomass comparatively, and a shift in the shape also indicates a structural difference. *A. thaliana* also has significant treatment differences for CLAI, with the control plant mean exceeding the *H. annuus* mean CLAI. However, prior to flowering *A. thaliana* does not grow vertical as *H. annuus* and *Z. mays*, so reflectance in the near-IR of ~0.3–0.4 in the FOV spectra relates more to biomass.

All species showed slightly increased reflectance in the FOV treatment group as compared to the control in week 1 (Figure 2). This implies a possible initial stimulatory effect at these time points, which is commonly seen response to low-level lithium exposure [7,47]. However, it also appears that there may be structural degradation at week 2 for all four species as treatment reflectance in FOV spectra is lower than control reflectance (Figure 2). In week 3 these differences remain in *A. thaliana* and *Z. mays* spectra but are no longer apparent in *H. annuus* or *B. napus* spectra. It has been suggested that plants could simultaneously experience both stimulatory effects and toxicity symptoms from Li exposure, due to different concentrations of Li within the plant [50,51]. A separate study saw stimulated growth along with slight chlorosis in leaves of snap bean (*Phaseolus vulgaris*) exposed to 4 ppm lithium nitrate (LiNO_3_) [47].

The vegetation indices that proved to be significant predictors of biomass for *A. thaliana* were YI with FOV and R_750_/R_550_ with both CP and FOV. Significant predictors of CLAI were NDVI, PSND, R_950_/R_750_, R_1636_/R_1933_, and (R_950_ − R_750_)/(R_950_ + R_750_) with FOV, and R_750_/R_550_ with both CP and FOV. NDVI has been well-associated with green biomass and leaf area [63]; here NDVI is not associated with *A. thaliana* biomass, but treatment-induced changes in biomass were also associated with necrosis and browning of the leaves. Biomass and CLAI are plant scale endpoints as opposed to leaf scale endpoints, however, R_750_/R_550_ was also a significant predictor of biomass at the leaf scale. One explanation for this is that biomass is related to leaf thickness (i.e., thinner leaves would result in less biomass), which would be seen in CP acquisition. Also, as statistically significant treatment effects were observed, *A. thaliana* biomass is related to lithium exposure. It is therefore highly likely that R_750_/R_550_ is responding to lithium uptake, particularly as it is a significant predictor of all other treatment-dependent endpoints considered. The remaining VI have been previously discussed. 

The significant predictors of *H. annuus* height were PSND, YI, and R_750_/R_550_, which are the same indices as were significant for chlorophyll content in *H. annuus* (Table 2). YI was significant with FOV spectra; as *H. annuus* leaves mottle slightly with time, the leaves also become broader and closer to the fore optics which could also be an increase in yellowness. PSND was significant with CP spectra, which is at first counterintuitive. However, as the plant grows taller, the internal structure of the plant changes as well. As discussed above, PSND has been associated with areal pigment concentration which likely changes as the plant grows. R_750_/R_550_ was significant at both the leaf and plant scales. *H. annuus* height has significant interaction effects, which essentially indicates that height differences depend on treatment duration. Lithium uptake into plants follows the dose given, although the specific relationship is species dependent [7]. Thus, height is likely associated (negatively) with lithium uptake. R_750_/R_550_, as above, is likely more indicative of lithium uptake than height specifically, as also discussed above for other endpoints.

## 5. Conclusions

This study explored the feasibility of using reflectance spectroscopy as a quick and easy-to-use technique in supplementing phytoremediation efforts. Certain vegetation indices seem promising for selected endpoints and species but the variable responses of plants to similar Li concentrations makes applying VI across species less reliable. Treating species with various concentrations of Li to induce a similar level of toxicity may be a more appropriate assessment of vegetation indices than assessing a certain level of Li across all species. For *A. thaliana*, R_750_/R_550_ was the best indicator of plant response to Li exposure, as it was a significant predictor of all endpoints considered, and it also has been previously associated with alkali metal (i.e., Cs) exposure [15,69]. Similarly, although with less predictive power, PSND, R_950_/R_750_, and R_1636_/R_1933_ were also previously associated with metal exposure [69], and would be reasonable VI to consider for identifying metal stress in the future. It is likely that a combination of VI and spectra collection techniques (i.e., CP and FOV) could provide the overall best approximation of plant stress status by accounting for both whole plant and leaf optical properties; multi-index use should be given future consideration in studies utilizing both CP and FOV.

Although we found some success in the laboratory for identifying relationships between symptoms induced by low-level Li exposure and reflectance spectra, environmental and sampling conditions were controlled; therefore, care should be given if the intent is to extrapolate to field studies. Measurements taken in the field may not be as consistent or informative as measurements taken in the laboratory due to extraneous and potentially unknown environmental factors. However, this study did find that the normalized difference (R_950_ − R_750_)/(R_950_ + R_750_) provided comparable results to its corresponding simple ratio in a situation with low background reflection. In another setting, utilization of normalized differences where simple ratios were useful here may still be fitting.

Reflectance spectra provide useful information, but they can be expected to be different for distinct species. However, when treatment reflectance spectra are considered relative to a control, stress may be able to be quantified across species. The general difference in metal toxicity symptoms between all four species explains why reflectance spectra did not always shift in similar ways. Knowledge of the mechanisms involved in plant species uptake and response to a desired metal is necessary to appropriately apply vegetation indices as predictors of stress. Consideration of individual reflectance spectra as well as treatment spectra relative to control can provide additional insight into understanding results.

## Figures and Tables

**Table 1:** Results (*p*-values) of initial statistical analysis of measured endpoints. Bold numbers indicate significance at the 0.05 level.

Species	Endpoint	Tmt (F(1,10))	Week (F(2,56))	Interaction (F(2,56))
*A. thaliana*	Chl *a + b*	**<0.0001**	**<0.0001**	**<0.0001**
	RWC	**<0.0001**	**<0.0001**	**<0.0001**
	Dry biomass	**0.0479**	**<0.0001**	**0.0014**
	CLAI	**<0.0001**	**0.0309**	**0.0002**
*Z. mays*	Chl *a + b*	0.1067	**<0.0001**	0.1922
	RWC	0.2580	**<0.0001**	0.4192
	Dry biomass	0.9923	**<0.0001**	0.5890
	CLAI	0.2652	0.4338	0.4271
*H. annuus*	Chl *a + b*	**0.0014**	**<0.0001**	0.2899
	RWC	0.9459	**<0.0001**	0.5909
	Dry biomass	0.4353	**<0.0001**	0.8903
	CLAI	0.3669	**0.0006**	0.7505
	Height	0.1703	**<0.0001**	**0.0031**
*B. napus*	Chl *a + b*	0.3459	0.1573	0.7325
	RWC	0.1600	**<0.0001**	0.5512
	Dry biomass	0.8766	**<0.0001**	0.0599
	CLAI	0.8302	**<0.0001**	0.4791

**Table 2:** Results of secondary mixed-effects analysis. Significant *p*-values shown in bold.

Endpoint	VI	*H. annuus*		*A. thaliana*	
		CP	FOV	CP	FOV
Chl *a + b*	NDVI	--	--	--	0.3617
	WI	--	--	0.8154	0.9284
	PSND	**0.0063**	--	**0.0200**	0.3836
	YI	--	**0.0202**	**0.0003**	0.1850
	R_950_/R_750_	--	--	0.0661	0.4488
	R_750_/R_550_	**<0.0001**	**0.0030**	**0.0205**	**0.0235**
	R_1636_/R_1933_	--	--	**0.0256**	0.7458
	(R_950_ − R_750_)/(R_950_ + R_750_)	--	--	0.0937	0.3938
RWC	NDVI	--	--	--	0.5406
	WI	--	--	0.9937	0.4283
	PSND	--	--	0.5333	0.7267
	YI	--	--	0.9289	0.5922
	R_950_/R_750_	--	--	**0.0257**	0.0632
	R_750_/R_550_	--	--	0.6446	**0.0194**
	R_1636_/R_1933_	--	--	0.3356	0.6893
	(R_950_ − R_750_)/(R_950_ + R_750_)	--	--	**0.0402**	0.0572
Dry biomass	NDVI	--	--	--	0.5192
	WI	--	--	0.1565	0.9351
	PSND	--	--	0.6989	0.5206
	YI	--	--	0.3900	**0.0007**
	R_950_/R_750_	--	--	0.7997	0.1980
	R_750_/R_550_	--	--	**<0.0023**	**<0.0001**
	R_1636_/R_1933_	--	--	0.5527	0.6381
	(R_950_ − R_750_)/(R_950_ + R_750_)	--	--	0.7680	0.1066
CLAI	NDVI	--	--	--	**0.0040**
	WI	--	--	0.5640	0.3781
	PSND	--	--	0.1282	**0.0058**
	YI	--	--	0.2448	0.9941
	R_950_/R_750_	--	--	0.4603	0.0663
	R_750_/R_550_	--	--	**0.0003**	**0.0415**
	R_1636_/R_1933_	--	--	0.2342	**0.0049**
	(R_950_ − R_750_)/(R_950_ + R_750_)	--	--	0.3739	**0.0266**
Height	NDVI	--	--	--	--
	WI	--	--	--	--
	PSND	**<0.0001**	--	--	--
	YI	--	**0.0229**	--	--
	R_950_/R_750_	--	--	--	--
	R_750_/R_550_	**<0.0001**	**<0.0001**	--	--
	R_1636_/R_1933_	--	--	--	--
	(R_950_ − R_750_)/(R_950_ + R_750_)	--	--	--	--

-- Not included in secondary analysis.

## Supplementary Materials

The following are available online at http://www.mdpi.com/1424-8220/18/9/2750/s1, Table S1: Average reflectance data acquired by FOV and CP.

## Appendix B

**Table A1:** Summary statistics for sample endpoints by week and treatment group. Cells contain the mean ± the standard deviation of the mean. For each cell n = 12, except dry biomass for *A. thaliana* for which n = 6 for each cell, and Chl *a* + *b* for *A. thaliana* in Week 3 for which n = 11 (insufficient plant material available for one sample).

Week	Group	Chl *a + b* (µg/mL)	RWC	Dry Biomass (mg)	CLAI
***Zea mays***					
1	Control	24.83 ± 2.30	0.916 ± 0.005	309.0 ± 102.1	0.371 ± 0.105
	Treatment	24.92 ± 2.76	0.915 ± 0.004	334.7 ± 93.5	0.304 ± 0.086
2	Control	25.34 ± 2.28	0.912 ± 0.005	502.4 ± 195.3	0.352 ± 0.084
	Treatment	26.79 ± 1.88	0.914 ± 0.005	552.2 ± 180.6	0.349 ± 0.077
3	Control	20.84 ± 4.21	0.902 ± 0.009	896.0 ± 368.0	0.378 ± 0.096
	Treatment	23.64 ± 2.12	0.905 ± 0.006	818.4 ± 285.5	0.364 ± 0.093
***Arabidopsis thaliana***					
1	Control	15.49 ± 2.18	0.928 ± 0.006	139.1 ± 27.8	0.563 ± 0.065
	Treatment	15.42 ± 1.33	0.918 ± 0.010	179.6 ± 77.7	0.505 ± 0.104
2	Control	18.35 ± 2.23	0.922 ± 0.010	264.4 ± 31.6	0.704 ± 0.067
	Treatment	15.47 ± 4.34	0.882 ± 0.012	183.4 ± 69.5	0.480 ± 0.109
3	Control	19.82 ± 2.93	0.916 ± 0.005	279.3 ± 25.1	0.730 ± 0.107
	Treatment	6.06 ± 3.40	0.629 ± 0.137	212.2 ± 69.1	0.461 ± 0.061
***Brassica napus***					
1	Control	13.66 ± 1.89	0.946 ± 0.003	271.0 ± 50.6	0.505 ± 0.068
	Treatment	13.67 ± 1.10	0.943 ± 0.004	313.9 ± 91.5	0.493 ± 0.068
2	Control	14.41 ± 1.13	0.943 ± 0.008	492.5 ± 146.4	0.617 ± 0.100
	Treatment	13.51 ± 2.13	0.934 ± 0.005	520.4 ± 112.6	0.602 ± 0.115
3	Control	15.12 ± 2.84	0.917 ± 0.018	924.7 ± 145.5	0.631 ± 0.080
	Treatment	14.45 ± 2.72	0.910 ± 0.019	831.6 ± 165.1	0.672 ± 0.095
***Helianthus annuus***					
1	Control	18.71 ± 2.35	0.932 ± 0.006	262.9 ± 130.6	0.427 ± 0.195
	Treatment	17.93 ± 3.24	0.928 ± 0.008	234.5 ± 113.2	0.398 ± 0.150
2	Control	23.92 ± 2.62	0.929 ± 0.008	397.7 ± 109.9	0.565 ± 0.137
	Treatment	20.73 ± 3.12	0.931 ± 0.007	345.8 ± 162.2	0.527 ± 0.152
3	Control	23.96 ± 2.88	0.911 ± 0.017	805.8 ± 201.3	0.651 ± 0.180
	Treatment	20.79 ± 3.74	0.913 ± 0.013	739.2 ± 218.2	0.556 ± 0.237

**Table A2:** Summary statistics for *H. annuus* height by week and treatment group. Cells contain the mean ± the standard deviation of the mean. For each cell, n = 12.

Week	Group	Height (cm)
1	Control	7.76 ± 2.04
	Treatment	8.89 ± 2.87
2	Control	11.91 ± 2.80
	Treatment	12.30 ± 3.60
3	Control	24.32 ± 4.63
	Treatment	18.86 ± 4.54

## Appendix C

**Table A3:** Results of initial mixed-effects analysis of VI. Significant *p*-values shown in bold.

Species	VI	CP			FOV		
		Tmt (F(1,10))	Week (F(2,56))	Interaction (F(2,56))	Tmt (F(1,10))	Week (F(2,56))	Interaction (F(2,56))
*H. annuus*	NDVI	--	--	--	0.2385	**0.0203**	0.0813
	WI	0.5019	**0.0069**	0.4049	0.3317	**0.0334**	0.8654
	PSND	**0.0295**	**0.0127**	0.8555	0.3109	**0.0217**	0.0746
	YI	0.1177	0.6125	0.4628	**0.0022**	**<0.0001**	**0.0079**
	R_950_/R_750_	0.3633	**<0.0001**	0.7413	0.7559	0.1525	0.2403
	R_750_/R_550_	**0.0071**	**<0.0001**	0.1573	**0.0008**	**<0.0001**	**0.0269**
	R_1636_/R_1933_	0.0653	0.8831	0.6997	0.8969	**<0.0001**	**0.0061**
	(R_950_ − R_750_)/(R_950_ + R_750_)	0.3618	**<0.0001**	0.7386	0.7619	0.1481	0.2351
*Z. mays*	NDVI	--	--	--	0.7568	**0.0006**	0.6193
	WI	0.6174	0.0843	0.4998	0.6413	**<0.0001**	0.9903
	PSND	0.2175	**0.0003**	0.0707	0.7104	**0.0005**	0.5628
	YI	0.4996	**0.0030**	0.1736	**0.0197**	**<0.0001**	0.0729
	R_950_/R_750_	0.4220	**<0.0001**	0.1904	0.9706	**<0.0001**	0.2914
	R_750_/R_550_	0.8550	**0.0002**	0.4873	0.1712	**0.0013**	0.7066
	R_1636_/R_1933_	0.3538	**0.0388**	0.2561	0.6007	0.1999	0.6718
	(R_950_ − R_750_)/(R_950_ + R_750_)	0.4221	**<0.0001**	0.1910	0.9879	**<0.0001**	0.2896
*B. napus*	NDVI	--	--	--	0.5029	**0.0004**	0.8694
	WI	**0.0103**	**<0.0001**	**0.0250**	0.1117	**0.0213**	0.6315
	PSND	0.1105	**<0.0001**	0.3902	0.4865	**0.0031**	0.9486
	YI	0.6543	**0.0027**	**0.0242**	0.5517	**<0.0001**	0.9030
	R_950_/R_750_	0.8266	**<0.0001**	0.1267	0.1847	**<0.0001**	0.1889
	R_750_/R_550_	0.3200	0.7560	**0.0431**	0.2639	**0.0001**	0.6164
	R_1636_/R_1933_	**0.0006**	**<0.0001**	0.0004	0.4581	**<0.0001**	0.6011
	(R_950_ − R_750_)/(R_950_ + R_750_)	0.8263	**<0.0001**	0.1276	0.1821	**<0.0001**	0.1806
*A. thaliana*	NDVI	--	--	--	**<0.0001**	**<0.0001**	**<0.0001**
	WI	**0.0003**	**<0.0001**	**<0.0001**	**0.0034**	**0.0060**	**<0.0001**
	PSND	**<0.0001**	**<0.0001**	**<0.0001**	**<0.0001**	**<0.0001**	**<0.0001**
	YI	**<0.0001**	**<0.0001**	**<0.0001**	**<0.0001**	**<0.0001**	**<0.0001**
	R_950_/R_750_	**<0.0001**	**<0.0001**	**<0.0001**	**<0.0001**	**<0.0001**	**<0.0001**
	R_750_/R_550_	**0.0001**	**<0.0001**	**<0.0001**	**<0.0001**	0.6830	**<0.0001**
	R_1636_/R_1933_	**<0.0001**	**<0.0001**	**<0.0001**	**<0.0001**	**<0.0001**	**<0.0001**
	(R_950_ − R_750_)/(R_950_ + R_750_)	**<0.0001**	**<0.0001**	**<0.0001**	**<0.0001**	**<0.0001**	**<0.0001**
